# Progress in Psychiatry

**Published:** 1901-02-02

**Authors:** 


### Progress in Psychiatry.

(*Continued from page* 51.)

**Toxic Action of Lead and Metallic Poisons.**—Lead has been found to produce in those exposed to its dust and fumes a special form of peripheral neuritis characterised by local paralysis of the extensors of the wrist (wrist-drop) as well as by the general symptoms of a profound general neuropathy. According to heredity and special predisposition the latter may appear as one or more forms of encephalopathy. Fifty years ago Tanquerel des Planches (*Thése de Paris*, 1848) pointed out that acute confusional insanity with delirium and hallucination may be produced by lead poisoning, and that the resulting mental (cerebral) degeneration may subsequently assume a chronic and incurable form clinically resembling paralytic dementia. In other cases the acute mental disease is succeeded by chronic epilepsy, and in some cases by a condition o mental irritability with delusions of persecution or of suspicion. This last condition may be compatible with long life, and the patient—a degenerate in mind—may live for years, marry and procreate offspring who are very prone to exhibit in some form or another the taint of degeneracy. Rennert had pointed out in 1882 that women employed in the pottery factories in Germany suffered from a form of lead poisoning which produced decidedly degenerative effects upon the offspring. "These women had frequent abortions, often produced deaf mutes, and very frequently macrocephalic children" (Talbot's *Degeneracy*, page 119).

Insanity among lead workers is found to be one of the chief dangers incurred by those exposed to the toxic action of the leaden ores. The trades most liable to the dangers are those of white and orange lead workers, painters and plumbers, workers in the manufacture of pottery, lead miners and smelters, and enamellers. Among the 1,050 male patients at the Claybury Lunatic Asylum there are, according to Dr. Robert Jones,[73] no fewer than 35 who have been workers in lead in some form of occupation, a fact which suggests *primâ facie* that the occupation of this class may be a contributory factor in the causation o insanity. The danger with lead begins with the smelting, the fumes given off by the molten metal being poisonous, and measures have been accordingly taken against inhaling the fumes by "hooding" the furnaces, or having high chimneys to remove the fumes. The greatest danger with lead is when the ores are put through the process of manufacture into "white lead" and "orange lead," both of which are highly poisonous owing to the presence of carbonate of lead, the fine dust of which produces, when inhaled or ingested, symptoms of poisoning. Lead is absorbed by the mucous membrane, and mainly through the mouth and nostrils. A case of lead poisoning with typical Aran-Duchenne paralysis is reported from washing the nose with a solution of acetate of lead for syphilitic erosion, and in a case reported by Dr. Savage lead poisoning resulted through the use of a vaginal douche. The skin when intact can aborb very small traces of lead in solution, and chronic lead poisoning has been stated to follow the use of hair washes and dyes containing lead. Lead absorbed into the system is stored in the liver, brain, and kidneys. Arterial degeneration, renal disease, anæmia, privation, and drink predispose *caeteris paribus* to lead toxæmia and lead encephalopathy. The young (aged 17 to 30 years) are more predisposed, especially women, but lead encephalopathy may occur in the middle-aged. A hereditary neurotic history predisposes to encephalopathy, as would any accident or injury. In 20 per cent. of Dr. Jones's cases with typical signs of lead poisoning, an injury such as a fall or kick was given as a predisposing cause.

The symptoms produced in animals by lead-poisoning are loss of appetite and rapid wasting, constipation (or diarrhœa with dark stools), a peculiar nervousness, albuminuria, and death. Pareplegia, loss of sexual power, and abortion have also been noticed after lead poisoning, and Vulpian has described the presence of myelitis. The special lead encephalopathy which affects the human subject includes a variety of mental and other cerebral symptoms. These comprise loss of memory, drowsiness, mental confusion or delirium with or without visual or aural hallucinations, excitability and other psychical disturbances. Loss of speech and blindness often follow lead encephalopathy, while epileptiform (and in females hysteriform) convulsions are very frequent. In 133 patients admitted to Claybury Asylum for insanity in which lead encephalopathy may have played a possible part, the mental conditions were as follows: 37 cases were cases of mania, 33 had melancholia, 24 had dementia with general paralysis, 19 had dementia, 10 had dementia with epilepsy, 7 had general paralysis, and 3 had alcoholic mania. "The proportion of general paralytics among these possible lead cases is 18 per cent. The average yearly percentage of general paralytics to the total average number of male patients admitted into asylums for the five years 1893–97 was 13·1 per cent. and it appears there is a strong presumptive evidence that lead may be a factor in the cause of general paralysis of the insane."[74] Several of the patients suffered from ataxiform symptoms; thus the gait is described as "ataxic" in two cases, shaky or jerky in three, feeble in two, fair in three, unsteady in one, good in two, normal in two, and not stated in four. The ataxia described in the above lead cases is probably not a true ataxia, although it may present a superficial resemblance, owing to partial anæsthesia of the soles of the feet. It is an inco-ordination due more to muscular weakness than to loss of muscular sense. The apparent jerkiness or jactitation in the gait may be due to a weakness of the flexors and an undue action of the extensors.

Alcohol is often associated with lead in the production of nervous symptoms, so that it becomes difficult to state what is due to the one and what to the other. Probably both lead and alcohol act directly as cerebral poisons. Moreover, the question of heredity in these cases requires to be considered. As regards diagnosis there are, apart from the somatic symptoms such as colic, blue line, wrist-drop, the special mental disturbances already referred to. Delusions of poisoning are frequent in lead encephalopathy, but delusions of grandeur and mental exaltation are less frequent. If the latter occur there is a co-extensive mental enfeeblement with listlessness, loss of memory and dementia. Cases of this kind tend to simulate general paralysis of the insane with a tendency to get well and thus are the cause of trouble and error in diagnosis. In a discussion at the recent meeting of the British Medical Association at Ipswich (August, 1900), Dr. Lloyd Andriezen[75] pointed out that lead encephalopathy is in some cases primary, that it is dependent on the direct action of lead salts on the nerve elements rather than secondary to uric acidæmia. The best proof of this was the fact that symptoms of encephalopathy, such as mental confusion, delirium and incoherence followed within a few hours of poisoning by lead. An instance was related of a family, members of which had eaten sweets containing lead chromate, with the result that several of them suffered from acute symptoms of cerebral toxæmia of the kind above mentioned, the symptoms appearing within 24 hours. At the same meeting Dr. H. Corner[76] pointed out that some cases of lead encephalopathy simulated acute alcoholism and occurred more frequently in young males, some of them having practically all the symptoms of delirium tremens, and others that of mania transitoria. Another class occurred amongst young girls and frequently simulated ordinary hysteria. Those cases that had epileptiform fits frequently ended fatally. Lead poisoning in children[77] has according to Hahn a special series of symptoms unlike those in adults. The effect is almost confined to the nervous system, and in no case have the symptoms of colic, anæmia, and the blue line on the gums been recorded. A child who was treated with Helva's ointment for eczema of the head developed symptoms of cerebral compression, became comatose, and died, and post-mortem analysis showed the presence of lead in considerable quantities in the brain. No case of poisoning from sucking leaden soldiers is said to have been as yet recorded.

Other metallic poisons such as mercury have been known to produce symptoms of encephalopathy and chronic toxæmia not unlike those from lead poisoning, but of much rarer occurrence.

[73] Brit. Med. Jour., Sept. 22, 1900. [74] Ibid., p. 797. [75] Ibid., p. 804. [76] Ibid., p. 804. [77] Archiv. fur Kinderheilkunde, Bd. xxviii. Hpt. 3.

